# Supplementary figures and images for: Incidence and predictors of mortality among persons receiving second-line tuberculosis treatment in sub-Saharan Africa: A meta-analysis of 43 cohort studies
Source: PLoS One. 2021 Dec 10;16(12):e0261149. doi: 10.1371/journal.pone.0261149 (PMC8664218; doi:10.1371/journal.pone.0261149)

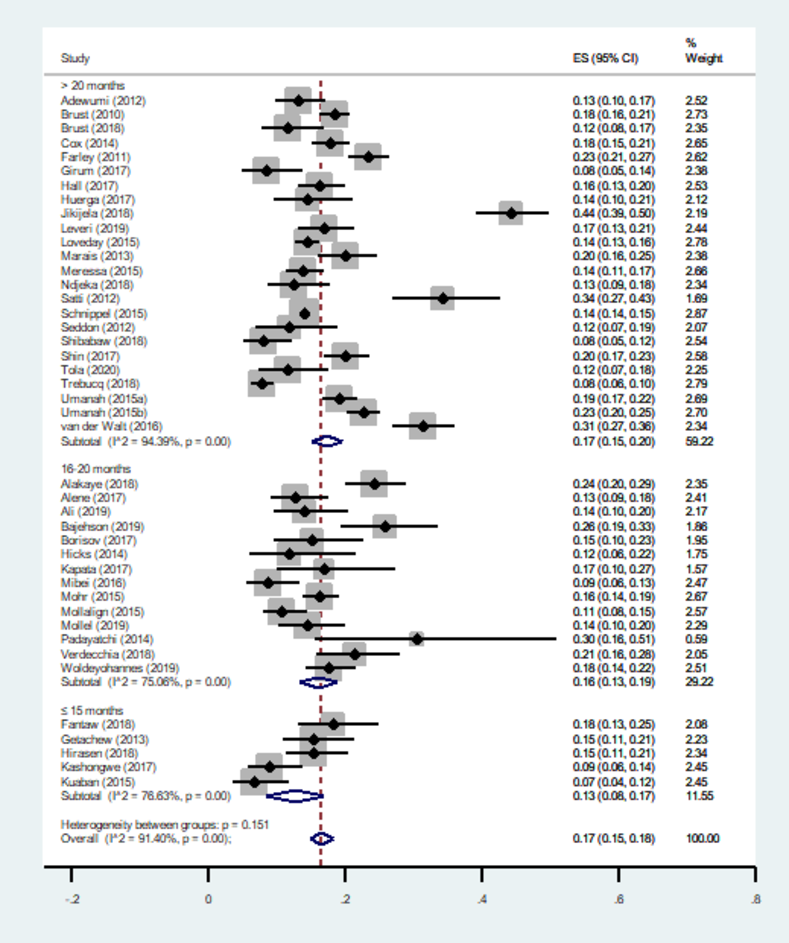

Supplement: S1 Fig — (TIF) [file pone.0261149.s001.tif]

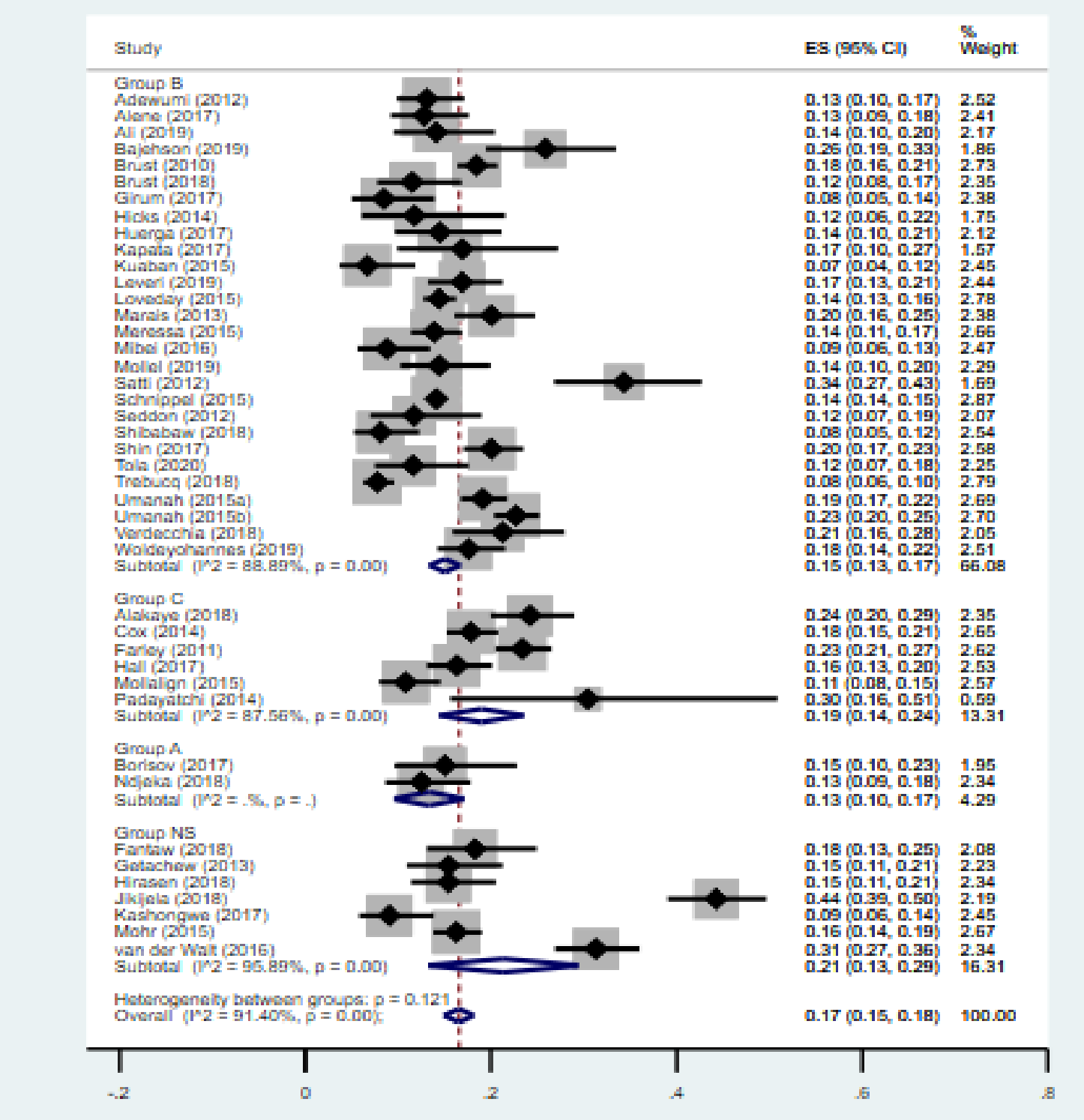

Supplement: S2 Fig — (TIF) [file pone.0261149.s002.tif]

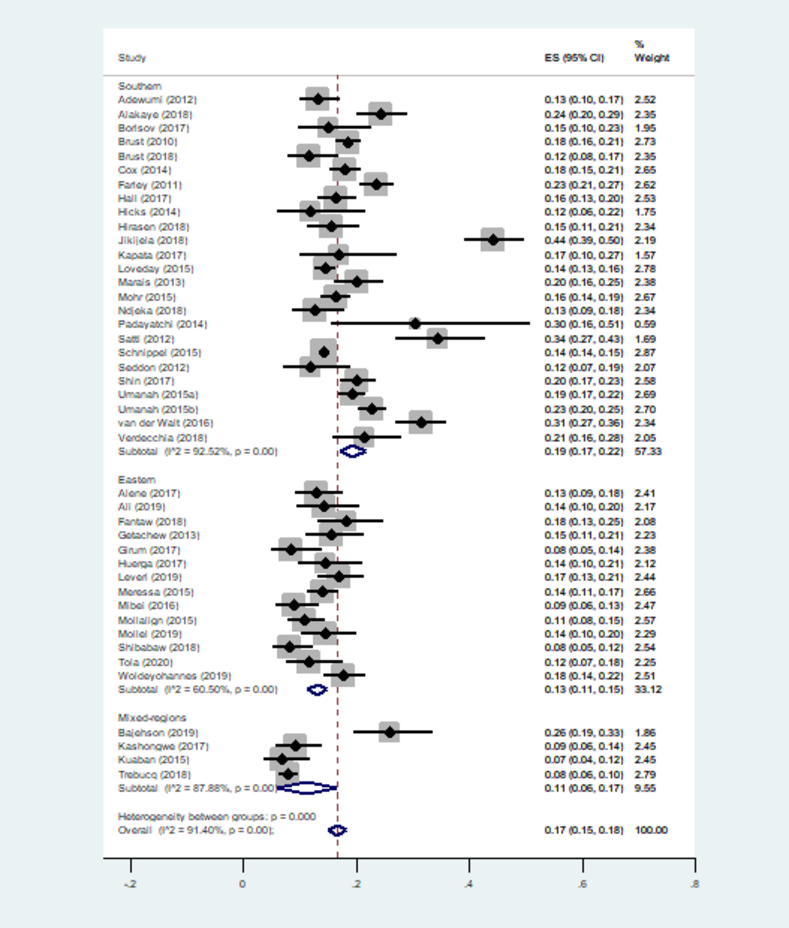

Supplement: S3 Fig — (TIF) [file pone.0261149.s003.tif]
