# Supplementary material for: Incidence and predictors of mortality among persons receiving second-line tuberculosis treatment in sub-Saharan Africa: A meta-analysis of 43 cohort studies
Source: PLoS One. 2021 Dec 10;16(12):e0261149. doi: 10.1371/journal.pone.0261149 (PMC8664218; doi:10.1371/journal.pone.0261149)
Supplement: S1 Table — (DOCX) [file pone.0261149.s004.docx]

**S1 Table: Search strategy and terms for PubMed, January 1, 2010-March 15, 2020.**

| **Search** | **Query** | **Result** |
| --- | --- | --- |
| 11 | ((((((second-line AND (rifampicin-resistant)) OR (multidrug-resistant)) AND (tuberculosis)) AND (treatment outcome)) OR (death)) AND (factors associated)) AND (Africa, South of the Sahara) | 1897 |
| 10 | Treatment outcome OR death | 1,164,974 |
| 9 | Rifampicin-resistant OR multidrug-resistant | 23,173 |
| 8 | Africa, South of the Sahara | 97,218 |
| 7 | Factors associated | 938,160 |
| 6 | Death | 448,444 |
| 5 | Treatment outcome | 777,959 |
| 4 | Tuberculosis | 73,267 |
| 3 | Multidrug-resistant | 22,865 |
| 2 | Rifampicin-resistant | 568 |
| 1 | Second-line* | 14,425 |
