# Supplementary material for: Incidence and predictors of mortality among persons receiving second-line tuberculosis treatment in sub-Saharan Africa: A meta-analysis of 43 cohort studies
Source: PLoS One. 2021 Dec 10;16(12):e0261149. doi: 10.1371/journal.pone.0261149 (PMC8664218; doi:10.1371/journal.pone.0261149)
Supplement: S2 Table — (DOCX) [file pone.0261149.s005.docx]

**S2 Table: Quality assessment for the included studies in the meta-analysis.**

| **References** | **JBI’s Critical Appraisal Checklist** | | | | | | | | | | | **Score (Yes)** |
| --- | --- | --- | --- | --- | --- | --- | --- | --- | --- | --- | --- | --- |
|  | **Q1** | **Q2** | **Q3** | **Q4** | **Q5** | **Q6** | **Q7** | **Q8** | **Q9** | **Q10** | **Q11** |  |
| Adewumi (2012) | Yes | NA | Yes | No | No | Yes | Yes | Yes | Yes | No | No | 6 |
| Alakaye (2018) | NA | NA | Yes | No | No | Yes | Yes | Yes | Yes | No | Yes | 6 |
| Alene (2017) | NA | NA | Yes | Yes | No | Yes | Yes | Yes | Yes | No | Yes | 7 |
| Ali (2019) | NA | NA | Yes | Yes | No | Yes | Yes | Yes | Yes | No | Yes | 7 |
| Bajehson (2019) | NA | NA | Yes | Yes | Yes | Yes | Yes | Yes | Yes | No | Yes | 8 |
| Borisov (2017) | NA | NA | Yes | No | No | Yes | Yes | Yes | Yes | No | Yes | 6 |
| Brust (2010) | NA | NA | Yes | Yes | No | Yes | Yes | Yes | No | No | Yes | 6 |
| Brust (2018) | NA | NA | Yes | Yes | No | Yes | Yes | Yes | Yes | No | Yes | 7 |
| Cox (2014) | NA | NA | Yes | Yes | No | Yes | Yes | Yes | Yes | Yes | Yes | 8 |
| Fantaw (2018) | NA | NA | Yes | Yes | No | Yes | Yes | Yes | No | No | Yes | 6 |
| Farley (2011) | Yes | UC | Yes | Yes | No | Yes | Yes | No | Yes | No | Yes | 7 |
| Getachew (2013) | NA | NA | Yes | Yes | No | Yes | Yes | Yes | No | No | Yes | 6 |
| Girum (2017) | NA | NA | Yes | Yes | No | Yes | Yes | Yes | Yes | No | Yes | 7 |
| Hall (2017) | NA | NA | Yes | Yes | No | Yes | Yes | Yes | Yes | No | Yes | 7 |
| Hicks (2014) | NA | NA | Yes | Yes | No | Yes | Yes | Yes | Yes | No | Yes | 7 |
| Hirasen (2018) | NA | NA | Yes | Yes | No | Yes | Yes | No | Yes | Yes | Yes | 7 |
| Huerga (2017) | NA | NA | Yes | Yes | No | Yes | No | Yes | Yes | No | Yes | 6 |
| Jikijela (2018) | NA | NA | Yes | Yes | No | Yes | Yes | Yes | No | No | Yes | 6 |
| Kapata (2017) | NA | NA | Yes | Yes | No | Yes | Yes | Yes | Yes | Yes | Yes | 8 |
| Kashongwe (2017) | NA | NA | Yes | Yes | No | Yes | Yes | Yes | No | No | Yes | 6 |
| Kuaban (2015) | NA | NA | Yes | Yes | No | Yes | Yes | Yes | No | No | Yes | 6 |
| Leveri (2019) | NA | NA | Yes | Yes | No | Yes | Yes | Yes | Yes | No | Yes | 7 |
| Loveday (2015) | Yes | UC | Yes | Yes | No | Yes | Yes | Yes | Yes | No | Yes | 8 |
| Marais (2013) | NA | NA | Yes | Yes | No | Yes | Yes | Yes | Yes | No | Yes | 7 |
| Meressa (2015) | NA | NA | Yes | Yes | Yes | No | Yes | Yes | Yes | No | Yes | 7 |
| Mibei (2016) | NA | NA | Yes | Yes | No | Yes | Yes | Yes | Yes | No | No | 6 |
| Mohr (2015) | NA | NA | Yes | Yes | No | Yes | Yes | Yes | Yes | No | Yes | 7 |
| Mollalign (2015) | NA | NA | Yes | Yes | No | Yes | Yes | Yes | Yes | No | Yes | 7 |
| Mollel (2019) | NA | NA | Yes | Yes | No | Yes | Yes | Yes | Yes | No | No | 6 |
| Ndjeka (2018) | NA | NA | Yes | Yes | Yes | Yes | Yes | Yes | Yes | Yes | Yes | 9 |
| Padayatchi (2014) | NA | NA | Yes | Yes | Yes | Yes | Yes | Yes | Yes | Yes | Yes | 9 |
| Satti (2012) | NA | NA | Yes | Yes | No | Yes | Yes | Yes | Yes | No | Yes | 7 |
| Schnippel (2015) | NA | NA | Yes | Yes | No | Yes | Yes | Yes | Yes | No | Yes | 7 |
| Seddon (2012) | NA | NA | Yes | Yes | No | Yes | Yes | Yes | Yes | No | Yes | 7 |
| Shibabaw (2018) | NA | NA | Yes | Yes | No | Yes | Yes | Yes | Yes | No | Yes | 7 |
| Shin (2017) | NA | NA | Yes | Yes | No | Yes | Yes | Yes | Yes | Yes | Yes | 8 |
| Tola (2020) | NA | NA | Yes | Yes | No | Yes | Yes | Yes | Yes | No | Yes | 7 |
| Trebucq (2018) | NA | NA | Yes | Yes | Yes | Yes | Yes | Yes | Yes | Yes | Yes | 9 |
| Umanah (2015a) | NA | NA | Yes | Yes | No | Yes | Yes | Yes | Yes | No | Yes | 7 |
| Umanah (2015b) | NA | NA | Yes | Yes | No | Yes | Yes | Yes | Yes | No | Yes | 7 |
| van der Walt (2016) | NA | NA | Yes | No | No | Yes | Yes | Yes | Yes | No | Yes | 6 |
| Verdecchia (2018) | NA | NA | Yes | Yes | No | Yes | Yes | Yes | Yes | No | Yes | 7 |
| Woldeyohannes (2019) | NA | NA | Yes | Yes | No | Yes | Yes | Yes | Yes | No | Yes | 7 |

**Note**: NA, not applicable; UN, unclear; Q1-11, JBI’s Critical Appraisal Checklist for Cohort studies {Q1: Were the two groups similar and recruited from the same population? Q2: Were the exposures measured similarly to assign people to both exposed and unexposed groups? Q3: Was the exposure measured in a valid and reliable way? Q4: Were confounding factors identified? Q5: Were strategies to deal with confounding factors stated? Q6: Were the groups/participants free of the outcome at the start of the study (or at the moment of exposure)? Q7: Were the outcomes measured in a valid and reliable way? Q8: Was the follow up time reported and sufficient to be long enough for outcomes to occur? Q9: Was follow up complete, and if not, were the reasons to loss to follow up described and explored? Q10: Were strategies to address incomplete follow up utilized? Q11: Was appropriate statistical analysis used?}.
