# Supplementary material for: Incidence and predictors of mortality among persons receiving second-line tuberculosis treatment in sub-Saharan Africa: A meta-analysis of 43 cohort studies
Source: PLoS One. 2021 Dec 10;16(12):e0261149. doi: 10.1371/journal.pone.0261149 (PMC8664218; doi:10.1371/journal.pone.0261149)
Supplement: Attachment — Submitted filename: PlosOne_Risk Factors of mortality_Review.docx [file pone.0261149.s007.docx]

| **Section of the paper** | **Comment** |
| --- | --- |
| *General* | |
| Content | I think that the authors need to highlight what makes this study novel as there have been similar systematic reviews and meta-analyses investigating factors associated with ones DR-TB prognosis, including mortaltiy. |
| Methodology | I generally think that the methodology used requires improvement. The inclusion criteria are not explicit and I think there is a possibility that patients have been included more than once in the analysis. Additionally, I think that the intent to treat methodology might have led to an underestimation of mortality. With that said I wonder how the risk can be accurately determined. |
| Editing | The authors should pay attention to the grammar used throughout. The grammar used needs improvement. The authors might benefit from an editing service to improve the structure and flow of many of these sections.  The authors should ensure that they define all abbreviated terms upon first time use and thereafter they can use the abbreviated term (i.e. SLD).  The authors should spell out numbers less than 10 unless they are linked to a unit of measurement. |
| *Title* | |
| Language and representativeness | The English used in the title is not clear. You might want to consider an alternative title for example:  Risk factors of mortality among persons receiving second-line tuberculosis treatment in sub-Saharan Africa: A meta-analysis of cohort studies  Additionally, in the title you might want to consider including the number of studies included in the meta-analysis. |
| *Abstract* | |
| Introduction | I think that the authors need to consider highlighting what makes this study novel here. |
| Methods | I think that the authors need to further expand on their inclusion and exclusion criteria here. |
| *Background* | |
| General | I recommend that you update the references to include the data from the WHO Global TB report from 2020.  Kindly clarify the language used after reference 6. I think you are referring to the missing TB cases. You could say something like in addition to the large number of missing incident DR-TB cases,….”  I recommend that you edit the first sentence of the second paragraph. The wording is quite awkward. In fact I recommend that this whole paragraph is edited in order to improve the flow and use of English language.  In the last paragraph, I think you need to focus more on what makes your study unique and novel as there have been various systematic reviews which have investigated the various DR-TB treatment correlates associated with outcomes. |
| *Methods* | |
| The protocol and registration | This paragraph requires editing for improved English language use. |
| Search strategy | I wonder why the authors did not use the term mortality in their search strategy? |
| Eligibility criteria | I am not sure what you mean by this? Do you mean you excluded studies that only evaluated treatment success? All DR-TB outcomes are intertwined. Even patients who are LTFU or whom fail treatment are at increased risk of mortality.  “But, we excluded records with outcomes unrelated or irrelevant to mortality and/or its risk factors.”  What do you define as insufficient as mentioned in the below comment? Additionally I am not sure as to your intended meaning when you say mixed patients? I am not sure how patients were excluded on these bases. This requires clarification to truly understand who was vs was not enrolled.  “Again, we excluded studies which report outcomes with insufficient quality and no separate result in the case of mixed patients from both extensively drug-resistant TB and multidrug-resistant TB treatments.”  I wonder if the authors only included studies which used the WHO definitions of outcomes.  Again, were only 24 month outcomes evaluated? Or were interim outcomes evaluated in this study. |
| Study selection procedure | If patients were retreatment cases, how did you decide which treatment episode to include in your study? |
| Data extraction process | As you say that you used intention to treat analysis, does this mean you included studies that reported on interim outcomes. Again, this requires clarification in the eligibility criteria section of the paper. |
| Outcomes definition  Page 4, line 36  Page 4, line 39 | Rather say “Outcome definitions”  The primary outcome in this study was the occurrence of AEs. |
| *Results* | |
| Study characteristics | I recommend that you do not say mixed-age patients. You could consider something like included both “adults and children”.  Why do you say 9 of the 34 included studies but then you also say that only 43 studies were included. |
| Table 1 | I see that you include patients with different follow-up durations i.e. you did not evaluate end of treatment outcomes and you including interim treatment outcomes. These studies might therefore underreport mortality. How did you account for this in your analysis?  There could be the same patients from the study conducted by Schnippel et al 2015 in the other South Africa based cohort studies as the study conducted by Schnippel reviewed the national treatment register. Have you ensured that you excluded duplicate records for all of these studies. |
| Risk Factors of Mortality | I see that the authors report on substance use as a risk factor for mortality but it would be helpful to have a better understanding of how this was quantified/classified in the included studies.  You say “experience of treatment failure” was a risk factor for mortality so did you evaluate all cause mortality in this study (i.e. mortality that also occurred after LTFU or Rx failure). This requires clarification in the methods section of the paper.  Later in this section you state the following:  “However, the risk of mortality was not significantly increased in under-five children, **in patients who failed treatment** and those patients who experienced ADE during course of the treatment.”  This seems to contradict the statement I mention in my above comment regarding treatment failure.  In your analysis of risk of mortality, did you look at all into the regimens received/the drugs included in the regimen and what drugs might have influenced mortality. Did you look at the cohort based on year of Rx initiation? Various factors have changed regarding policy and clinical practice from 2010-current and therefore I wonder how the authors accounted for the change in the management of the disease over time.  I wonder if there were any social factors you could assess as well in order to determine risk factors associated with mortality. i.e. household income, female led households, number of people in the household, setting/housing structure |
| *Discussion* | |
| First paragraph | I recommend that you first summarize your finding without yet making reference to other studies in detail. Additionally, I would not say that the mortality is justified because then it sounds as if we are saying that it is “OK” that the mortality for these individuals is acceptable. We might be able to explain the factors contributing to it but it is far from acceptable and no where in line with making progress towards achieving the goals outlined in the EndTB strategy.  You could just generally compare your mortality rate to that reported by the WHO for all patients enrolled on treatment globally as a baseline and then delve more specifically into country level mortality. |
| General | I think this section requires many improvements. It seems that you just compare your findings to those from previously published studies rather than really looking into the policy and implementation factors that might be driving these risk factors. You might want to consider mentioning different models of care or approaches to TB control and management that can be employed to mitigate these risk factors, which have already been well documented in the literature. I do not think that the risk factors you point to here are different in any way from those already known and well documented in the literature. |
| *Limitations* | |
| General comments | I do not think that the intention to treat approach is realistic to procure good enough evidence in this study to inform policy regarding mortality. Without systematic follow-up for everyone mortality could be underestimated and the identified risk could therefore be less than that in reality should we have complete follow-up for this cohort of patients. |
| *Conclusions* | |
| General comments | I feel as though this section is weak. Although you have summarized your key findings and made recommendations they are very general. I think you could have better utilized the discussion section to expand on the various measures that could be employed to address high rates of mortality. |
| *Figures* | |
| General comment | Ensures that your figure quality is improved as the figures are blurry. |
