# Supplementary material for: Incidence and predictors of mortality among persons receiving second-line tuberculosis treatment in sub-Saharan Africa: A meta-analysis of 43 cohort studies
Source: PLoS One. 2021 Dec 10;16(12):e0261149. doi: 10.1371/journal.pone.0261149 (PMC8664218; doi:10.1371/journal.pone.0261149)
Supplement: Attachment — Submitted filename: Rebuttal letter to reviewers comments.docx [file pone.0261149.s008.docx]

**Authors’ rebuttal letter to reviewers’ comments**

PONE-D-20-25643
Risk factors of mortality during course of second-line tuberculosis treatment in sub-Saharan Africa: A meta-analysis of cohort studies
PLOS ONE

Journal Requirements:

When submitting your revision, we need you to address these additional requirements.

 1. Please ensure that your manuscript meets PLOS ONE's style requirements, including those for file naming. The PLOS ONE style templates can be found at

https://journals.plos.org/plosone/s/file?id=wjVg/PLOSOne_formatting_sample_main_body.pdf and

<https://journals.plos.org/plosone/s/file?id=ba62/PLOSOne_formatting_sample_title_authors_affiliations.pdf>

Response: This is addressed for all files we included to the main manuscript and supplementary materials.

2.We suggest you thoroughly copyedit your manuscript for language usage, spelling, and grammar. If you do not know anyone who can help you do this, you may wish to consider employing a professional scientific editing service.  

Whilst you may use any professional scientific editing service of your choice, PLOS has partnered with both American Journal Experts (AJE) and Editage to provide discounted services to PLOS authors. Both organizations have experience helping authors meet PLOS guidelines and can provide language editing, translation, manuscript formatting, and figure formatting to ensure your manuscript meets our submission guidelines. To take advantage of our partnership with AJE, visit the AJE website (http://learn.aje.com/plos/) for a 15% discount off AJE services. To take advantage of our partnership with Editage, visit the Editage website (www.editage.com) and enter referral code PLOSEDIT for a 15% discount off Editage services.  If the PLOS editorial team finds any language issues in text that either AJE or Editage has edited, the service provider will re-edit the text for free.

Response: We don’t have serious problem with English language usage. We strictly revised the manuscript and did grammar and language edition thoroughly. Again, a native English speaker also made edition to the revised manuscript. This professional is acknowledged in acknowledgement section the manuscript. So, the manuscript is now improved for English language usage problems. Please check the revised manuscript.

Upon resubmission, please provide the following:

- The name of the colleague or the details of the professional service that edited your manuscript
- A copy of your manuscript showing your changes by either highlighting them or using track changes (uploaded as a *supporting information* file)
- A clean copy of the edited manuscript (uploaded as the new *manuscript* file)

 3. Please confirm that you have included all items recommended in the PRISMA checklist including the full electronic boolean search strategy used to identify studies with all search terms and limits for at least one database. Please attach this as supplementary file

Response: We have added a supplementary file for search results and Boolean search strategy of PubMed as S1 Table in supporting information section.

4. In your methods section, please provide the dates when you accessed the data used for your study (it appears to be February to March 2020), as well as the publication date range that was used to search for the manuscripts included in your study.

Response: We conducted search for our analysis from February 1, 2020 to March 15, 2020. And, the publication date for our inclusion was from January 2010 to March 15, 2020. These same descriptions are included in search strategy and eligibility criteria of methods section in the revised manuscript.

Reviewers' comments:

Reviewer #1: Section of the paper Comment

| **Section of the paper** | **Comment** |
| --- | --- |
| *General* | |
| Content | I think that the authors need to highlight what makes this study novel as there have been similar systematic reviews and meta-analyses investigating factors associated with ones DR-TB prognosis, including mortality.  Authors’ response: Thank you. There were so many changes to treatment strategy and approaches for drug-resistant TB (DR-TB) in the last decade. The EndTB strategy was one the initiation of the last decade. In this regard, novel drugs (bedaquiline, delamanid), repurposed drugs (linezolid, clofazimine), consideration of later generation flouroquinolones (levofloxacin, moxifloxacin, gatifloxacin), and addition of these agents to standardized second-line anti-tuberculosis (SLD) therapy to make regimens shorter and more effective regimens were essential inputs of the last decade. So, analyzing mortality benefits of these approaches to DR-TB patients is very essential for alerting whether we are on the way to achieving the EndTB strategy target or not. These explanations are indicated in introduction section of the revised manuscript as an update. |
| Methodology | I generally think that the methodology used requires improvement. The inclusion criteria are not explicit and I think there is a possibility that patients have been included more than once in the analysis. Additionally, I think that the intent to treat methodology might have led to an underestimation of mortality. With that said I wonder how the risk can be accurately determined.  Authors’ response: We have revised our methodology in response to your recommendation. The eligibility criteria also adds exclusion of Pre-XDR and XDR-TB as these could be part of unfavorable outcomes for RR/MDR-TB treatment. Again, there could also be aggressive treatment of such patients with changing and substitution of individual drugs in the regimen. This can complicate the outcome observed for a specific SLD regimen. By the intent-to treat principle, we considered all death reports as all-cause mortality for all studies including those which report interim outcomes. We accounted for this with our subgroup analysis by the median month of SLD therapy. However, we found no significant difference in the mortality estimates by subgrouping (See S1 Fig). |
| Editing | The authors should pay attention to the grammar used throughout. The grammar used needs improvement. The authors might benefit from an editing service to improve the structure and flow of many of these sections.  The authors should ensure that they define all abbreviated terms upon first time use and thereafter they can use the abbreviated term (i.e. SLD).  The authors should spell out numbers less than 10 unless they are linked to a unit of measurement.  Authors’ response: The English grammar used throughout is edited and corrected in the revised manuscript. The structure and flow of the paragraphs are also improved in the revised manuscript. Moreover, all abbreviated terms are fully described first for repeated use thereafter. |
| *Title* | |
| Language and representativeness | The English used in the title is not clear. You might want to consider an alternative title for example:  Risk factors of mortality among persons receiving second-line tuberculosis treatment in sub-Saharan Africa: A meta-analysis of cohort studies  Additionally, in the title you might want to consider including the number of studies included in the meta-analysis.  Authors’ response: Thank you so much. These comments are fully addressed in the revised manuscript. |
| *Abstract* | |
| Introduction | I think that the authors need to consider highlighting what makes this study novel here.  Authors’ response: We have highlighted all relevant information that made the current meta-analysis novel in the introduction section of the revised manuscript. Please see also our response to content section of your general comment. |
| Methods | I think that the authors need to further expand on their inclusion and exclusion criteria here.  Authors’ response: Descriptions we modified in eligibility section of the revised manuscript include publication dates for included studies (Jan 2010 to Mar 15, 2020), consideration of studies which report either complete or interim outcome. We also excluded studies which enrolled Pre-XDR and XDR-TB patients and MDR and XDR-TB patients with no separate outcome report for the MDR-TB patients. |
| *Background* | |
| General | I recommend that you update the references to include the data from the WHO Global TB report from 2020.  Kindly clarify the language used after reference 6. I think you are referring to the missing TB cases. You could say something like in addition to the large number of missing incident DR-TB cases,….”  I recommend that you edit the first sentence of the second paragraph. The wording is quite awkward. In fact I recommend that this whole paragraph is edited in order to improve the flow and use of English language.  In the last paragraph, I think you need to focus more on what makes your study unique and novel as there have been various systematic reviews which have investigated the various DR-TB treatment correlates associated with outcomes.  Authors’ response: Thank you so much for these essential inputs. All the comments are accepted and modifications are made in introduction section of the revised manuscript. The WHO report data regarding DR-TB is also updated for 2020. We thought this analysis is an important feedback for evaluating impacts of advances, efforts and treatment approaches and modifications made in the last decade for DR-TB treatment that can alert whether we are on the way to meeting the set target by the EndTB strategy. The last decade evolution included addition of novel drugs, and repurposed agents to standardized SLD regimen and shortening of some of the SLD regimen. All these issues and others are added to the introduction section as justification to our current meta-analysis requirement. |
| *Methods* | |
| The protocol and registration | This paragraph requires editing for improved English language use.  Authors’ response: The English language usage for this paragraph in the revised manuscript is improved. Please check the protocol and its registration section of the methods. |
| Search strategy | I wonder why the authors did not use the term mortality in their search strategy?  Authors’ response: Thank you. But, death is also a medical subject heading (MeSH) term for mortality in our PubMed search for mortality. |
| Eligibility criteria | I am not sure what you mean by this? Do you mean you excluded studies that only evaluated treatment success? All DR-TB outcomes are intertwined. Even patients who are LTFU or whom fail treatment are at increased risk of mortality.  “But, we excluded records with outcomes unrelated or irrelevant to mortality and/or its risk factors.”  Authors’ response: These exclusion criteria specifically focuses on outcome such as additional resistant mutations to a drug in SLD regimen without mortality reports. Again, we mean outcome reported for a drug substituted in SLD for which additional and extended resistance could be the case. These issues can distort the mortality outcome of RR/MDR-TB considerations for this study. Moreover, even though there could be deaths from among LTFU and treatment failed patients, we can’t adjust mortality in such outcomes unless adjusted and reported by authors. This drawback is acknowledged in limitation section of the discussion section.    What do you define as insufficient as mentioned in the below comment? Additionally I am not sure as to your intended meaning when you say mixed patients? I am not sure how patients were excluded on these bases. This requires clarification to truly understand who was vs was not enrolled.  “Again, we excluded studies which report outcomes with insufficient quality and no separate result in the case of mixed patients from both extensively drug-resistant TB and multidrug-resistant TB treatments.”  Authors’ response: We include and exclude studies based on some criteria, but not patients. Based on the methodological quality assessment using JBI critical appraisal checklist, studies with average positive evaluations less than 50% for the overall criteria were not considered. Again, our focus was DR-TB (RR-TB and MDR-TB) and we should get a separate outcome data for RR/MDR-TB in case of studies which participated patients from XDR-TB and DR-TB as well. Some of the studies we excluded with reason of insufficient outcome report outcomes using mathematical model only. Again, some of them report deaths by simple observation in very small number (size) of patients without an appropriate methodology for that study. Such studies did not meet our eligibility criteria for quality and sufficiency of the data reported and thus excluded.  I wonder if the authors only included studies which used the WHO definitions of outcomes.  Authors’ response: WHO definitions of outcomes include treatment success, mortality, LTFU, and treatment failure. So, from these outcomes we focused on mortality outcome and risk factors correlated with it.  Again, were only 24 month outcomes evaluated? Or were interim outcomes evaluated in this study.  Authors’ response: For this study, death report for interim outcome was also considered to account for the real scenario of patient treatments and outcomes. So, death report for RR/MDR-TB patients noted after SLD initiation by the studies was considered irrespective of median month of the SLD therapy and death occurrence during the treatment. We accounted for this by our subgroup analyses by treatment duration. |
| Study selection procedure | If patients were retreatment cases, how did you decide which treatment episode to include in your study?  Authors’ response: Our consideration in this regard was primary RR/MDR-TB which can be ensured by the type of treatment the patients used. So, SLD therapy recommended by WHO for primary RR-TB and MDR-TB were at the center of our decision. There also was no reason to exclude retreatment cases for patients initiated with SLD regimen. |
| Data extraction process | As you say that you used intention to treat analysis, does this mean you included studies that reported on interim outcomes. Again, this requires clarification in the eligibility criteria section of the paper.  Authors’ response: Yes, it was not our intension to do so, but few studies reported the mortality outcome just as they occurred. Whatever the case, mortality outcome should be reported for patients who completed or did not complete the treatment. But, since we are reporting comprehensive mortality estimate pooled from 43 studies included, we don’t have space to adjust for mortality from LTFU and failed treatment. Thus, the mortality considered was all-cause mortality. And, this recommendation is addressed in methods section of the revised manuscript. |
| Outcomes definition  Page 4, line 36  Page 4, line 39 | Rather say “Outcome definitions”  The primary outcome in this study was the occurrence of AEs.  Authors’ response: This section comment doesn’t apply to our manuscript as we don’t have a subsection entitled with ‘outcomes definition’. However, we have included a subsection entitled as ‘outcome definitions’ in response to the other reviewer’s comment. |
| *Results* | |
| Study characteristics | I recommend that you do not say mixed-age patients. You could consider something like included both “adults and children”.  Why do you say 9 of the 34 included studies but then you also say that only 43 studies were included.  Authors’ response: Thank you. We thought this as there was no age preference in the studies and we were unable to classify them as children, adolescent or adults. So, in response to your comment, we have considered them as ‘adults and children’ for such subjects in our current revision.  For the number dealing with 9, we were saying 9 out of 43 of the included studies had a prospective cohort design while the design for the remaining 34 studies was retrospective cohort. This comment is clearly presented in the current revision. |
| Table 1 | I see that you include patients with different follow-up durations i.e. you did not evaluate end of treatment outcomes and you including interim treatment outcomes. These studies might therefore underreport mortality. How did you account for this in your analysis?  Authors’ response: The different follow-up duration presented was a median time of treatment the included studies had already reported. The mortality outcome for some of the studies included could also be interim outcome. That means, this could have been captured at any time after treatment initiation before completing treatment duration expected for standardized SLD regimen. However, some newer anti-TB drugs approved by WHO in the last decade could shorten the duration of SLD regimens from 24 or 18 months to below these periods.  In analysis stage, this difference was accounted for by subgroup analyses. In this regard, it was ranged from 13% (95% CI: 8-17%) for studies with median duration of treatment below 15 months to 18% (95% CI: 13-19%) for studies with median duration of therapy between 16 and 20 months. As it can be seen from the mortality estimates, the difference estimated was not statistically significant. This subgroup analysis estimate for mortality by median duration of SLD treatment is added to supplementary files.  There could be the same patients from the study conducted by Schnippel et al 2015 in the other South Africa based cohort studies as the study conducted by Schnippel reviewed the national treatment register. Have you ensured that you excluded duplicate records for all of these studies.  Authors’ response: Thank you for this critical concern. Yes, time of data capturing for Cox et al, 2014, Loveday et al, 2015, Mohr et al 2015 and Schnippel et al 2015 look overlapped to some extent. The remaining studies conducted in South Africa had a different data collection period. The Schnippel et al 2015 study was based on an electronic National DR-TB register program. But, the other studies report outcome for community-based prospective and retrospective programs. For the Schnippel et al 2015 study, there was no explicit descriptions of settings considered. So, we are not sure whether these studies have considered the same patients or not in order for us to avoid duplicates without knowledge of data source settings included in the register. |
| Risk Factors of Mortality | I see that the authors report on substance use as a risk factor for mortality but it would be helpful to have a better understanding of how this was quantified/classified in the included studies.  Authors’ response: Yes, some of the included studies report smoking and alcohol use as correlates of mortality. However, none of them clearly indicated how patient’s use of the substances was quantified. They narrate as excessive uses of alcohol and cigarette smoke. But, they report the odds or risks of these uses without categorization of uses as excessive, misuse, abuse, addicted, etc.  You say “experience of treatment failure” was a risk factor for mortality so did you evaluate all cause mortality in this study (i.e. mortality that also occurred after LTFU or Rx failure). This requires clarification in the methods section of the paper.  Authors’ response: Yes, the mortality reported was not attributable to any specific factor and it is an all-cause mortality. This is clearly presented in the current revision of the manuscript.  Later in this section you state the following:  “However, the risk of mortality was not significantly increased in under-five children, **in patients who failed treatment** and those patients who experienced ADE during course of the treatment.”  This seems to contradict the statement I mention in my above comment regarding treatment failure.  Authors’ response: Yes, the first paragraph was a study characteristic description for studies linking mortality with correlated factors. But, since our pooled estimate for RR of mortality in previous submission combined values only, we couldn’t indicate specific studies which report each factor. Accordingly, we revised our pooled estimate analysis for three or more studies reporting about a risk factor. So, we qualitatively narrate reports of the at most two studies linking mortality and a factor. Hence, the first description we explained as risk factors of mortality for all factors justified were those factors identified and reported by the studies we included. Our estimates follow in the next paragraph and some of the factors did not have link with mortality in final pooled estimate of RR.  In your analysis of risk of mortality, did you look at all into the regimens received/the drugs included in the regimen and what drugs might have influenced mortality. Did you look at the cohort based on year of Rx initiation? Various factors have changed regarding policy and clinical practice from 2010-current and therefore I wonder how the authors accounted for the change in the management of the disease over time.  Authors’ response: Yes, but there was no significant difference by WHO grouping of SLD regimen used by patients. The mortality was 13% (95% CI: 10-17%) for Group A, 15% (95% CI: 13-17%) for group B, 19% (95% CI: 14-24%) for group C and 21% (95% CI: 13-29%) for SLD regimen group not specified (S2 Fig). These values can give hints of reduced mortality for DR-TB patients treated with group A and B. These groups of SLD regimen are part of advances and modifications in DR-TB therapy in the last decade including treatment with novel drugs (bedaquiline, delamanid), repurposed agents (linezolid, clofazimine), later generation flouroquinolones (moxifloxacin, levofloxacin), and shorter regimens.    I wonder if there were any social factors you could assess as well in order to determine risk factors associated with mortality. i.e. household income, female led households, number of people in the household, setting/housing structure  Authors’ response: Since this is a meta-analysis of studies which report factors which have link with mortality outcome, we cannot select factors by ourselves. This is so because we don’t have complete individual patient data report of the studies. But, in our data extraction process we didn’t get studies that link social factors to mortality outcome. |
| **Discussion* | |
| First paragraph | I recommend that you first summarize your finding without yet making reference to other studies in detail. Additionally, I would not say that the mortality is justified because then it sounds as if we are saying that it is “OK” that the mortality for these individuals is acceptable. We might be able to explain the factors contributing to it but it is far from acceptable and no where in line with making progress towards achieving the goals outlined in the EndTB strategy.  You could just generally compare your mortality rate to that reported by the WHO for all patients enrolled on treatment globally as a baseline and then delve more specifically into country level mortality.  Authors’ response: These comments are fully addressed in the revised manuscript. Please check the discussion section of the manuscript. |
| General | I think this section requires many improvements. It seems that you just compare your findings to those from previously published studies rather than really looking into the policy and implementation factors that might be driving these risk factors. You might want to consider mentioning different models of care or approaches to TB control and management that can be employed to mitigate these risk factors, which have already been well documented in the literature. I do not think that the risk factors you point to here are different in any way from those already known and well documented in the literature.  Authors’ response: We considered your recommendation in the current revision and report overall estimate of the factors contributing to mortality which can be considered as acceptable as per the EndTB Strategy target set. This could be a result of the several efforts and approaches to DR-TB treatment in the last decade. |
| *Limitations* | |
| General comments | I do not think that the intention to treat approach is realistic to procure good enough evidence in this study to inform policy regarding mortality. Without systematic follow-up for everyone mortality could be underestimated and the identified risk could therefore be less than that in reality should we have complete follow-up for this cohort of patients.  Authors’ response: Yes, it doesn’t be a realistic approach for capturing enough evidence for mortality among patents put on treatment, but the mortality occurred at any time after treatment initiation need to be quantified. This means that the studies included report mortality at any time irrespective of the regimen completion or not. Such mortality they reported may not be attributable to failure of SLD regimen and it should be considered as all-cause mortality. In real practice, patients may drop regimen doses after taking some doses or may become mortal while taking medications before completing the regimen. So, we thought our consideration to the intention-to-treat approach description in limitation section was a reflection to the above description. |
| *Conclusions* | |
| General comments | I feel as though this section is weak. Although you have summarized your key findings and made recommendations they are very general. I think you could have better utilized the discussion section to expand on the various measures that could be employed to address high rates of mortality.  Authors’ response: Thank you for this concern. We have made modifications to the conclusion section of the revised manuscript. Please check this. |
| *Figures* | |
| General comment | Ensures that your figure quality is improved as the figures are blurry.  Authors’ response: Since the number of studies included for the meta-analysis was 43, we failed to enhance visibility of our fig 2. But, we tried to improve it in the current revision using the PLOS’s Apex.PACE. Please check it. |

Reviewer #2: In this meta-analysis the authors set out to identify risk factors for mortality during treatment for RR/MDR-TB in sub-Saharan Africa. The central findings of the analysis include the following: 17% of patients died during RR/MDR-TB treatment, and risk factors included low BMI (RR 2.5), substance use (or abuse?) (RR 2.5), HIV (RR 2.2), co-morbid conditions (RR 2.0), resistance to SLD (RR 1.92), anemia (RR 1.75), male sex (RR1.8) and delayed initiation of RR/MDR treatment for > 1 month (RR1.6). The methodology appears scientifically sound however this manuscript requires professional language and grammatical editing prior to publication

Authors’ response: Since our previous estimate reports risk factors for aggregate factors, pooling for individual risk factors is currently required and has brought slight value changes to RR values we estimated in previous submission. In this regard, we left a pooled estimate for factors reported by less than three studies. Instead, we narrated these factors in reference to the link between mortality and the factor reported by the included study. At the end, low baseline BMI (RR 2.79), substance use (RR 2.78), HIV-infection (RR 1.64), co-morbid conditions (2.75), resistance to SLD (RR 1.75), anemia (RR 1.90), and male sex (RR 1.82) were key factors estimated for pooled RR and found to have significant link with the mortality outcome.

We also corrected language usage gaps for the whole document; this can be seen in the revised manuscript.

Introduction
The introduction cites outdated references and incorrect statistics.
- (page 3) “As a result, only near to half of the drug-resistant TB patients are successfully treated; with approximately up to 40% of them dying during the treatment” – this statement is inaccurate. According to the WHO 2020 Global TB report, for the 2017 global cohort, 57% of RR/MDR-TB patients completed treatment successfully, 15% died, 16% were lost to follow-up and 7% failed. These figures have remained fairly stable for several years (see WHO Global TB report 2020 page 107).
- (page 3) “It (mortality) was alarmingly increased to over 30-40 percent in resource-limited settings”. This is not an accurate reflection of the WHO data on RR/MDR-TB mortality in sub-Saharan Africa where treatment success rates and mortality mirror the global averages. If the authors are including in this statement people who die without accessing treatment (undiagnosed or untreated) then they should clarify this sentence.
- Reference #2 and 5: Should cite updated statistics from the WHO 2020 global health report (introduction).

Authors’ response: Thank you for all the updates. These updates are included in introduction section of the revised manuscript. We considered current data from WHO 2020 global TB report as you suggested. Actually, some of the figures we presented in this section were estimation for cases detected and undetected.

Methods:
- What search terms were used? The authors should strongly consider including a table describing the search terms used in the study in a supplementary appendix

Authors’ response: The search terms used were described in search strategy section of the methods. A similar content with Boolean use for PubMed search strategy is added to a supplementary file in supporting information section revised manuscript that can describe our search terms.

- Was this an individual patient meta-analysis or aggregate data meta-analysis? This should be stated.

Authors’ response: It was an aggregate data meta-analysis for which we searched for published studies and extracted their data to give a summary (pooled) estimate. In this regard, since we have very limited control over the data, we tried to reduce heterogeneity by searching for unpublished studies from repositories of some academic institution.
- Restrictions on studies: the authors only mention dates and English language as restrictions, however based on the final selection there must have been other restrictions. Possible restrictions that the authors don’t mention but that would have factored into their selection include:
o Exclusion of clinical trials
o Limiting the studies to patients with rifampicin-resistant and/or multi-drug-resistant TB
o Were reports of pre-XDR/XDR of FQ-R TB excluded or included?

Authors’ response: Actually, we didn’t exclude clinical trials, but majority of them were multi-centered and separate data for SSA with regard to mortality outcome were not presented by the studies. Anyways, no study was excluded on ground of its clinical trial design. However, we excluded Pre-XDR/XDR-TB as this could be one of the unfavorable outcome to SLD therapy. Once Pre-XDR/ XDR-TB was detected, it has to be aggressively treated by individual drug modification, substitution, and addition of other agents than the initial SLD. This complicates and distorts the outcome observed after a SLD regimen initiation. This explanation is indicated in methods section of the revised manuscript.

- How many investigators extracted the data? Was it done concurrently or separately and were differences in data extraction reviewed?
- What data was extracted from the reviewed studies?

Authors’ response: Two of the investigators (DE and FA) extracted the data independently. They finally discussed together to reach on consensus regarding the values and variables of data extracted. The data we extracted include name of first author, year of publication, number of patients died during SDL treatment, total number of patients treated with SLD (sample size), median treatment duration for SLDs used, study design, study setting, age category of patients, group of SLD regimen used, and specific SLD regimen used if available. Again, we extracted factors the studies have linked with mortality outcome. They include HIV-coinfection, anemia, low BMI, resistance to SLD, co-morbid conditions, substance use, male sex, etc. These descriptions are clearly presented in data extraction process of the methods section in the revised manuscript.

- How was substance abuse/use defined in the studies that evaluated this as a risk factor for mortality?

Authors’ response: the substance use quantification of the studies was not clear, but they linked excessive use of alcohol and cigarette smoke to mortality outcome among the patients treated with SLD regimen.
- How was second-line drug-resistance defined (i.e. resistance to aminoglycosides and/or FQs) in the studies that included this data? And what were the proportions of patients with AG vs FQ resistance in those included in the review.

Authors’ response: Resistance to SLD was defined as a resistance to at least three anti-TB drugs. A resistance to any fluoroquinolone (Cox et al, 2014) and, at least one SLD from injectable aminoglycosides (kanamycin, amikacin, capreomycin) (Schnippel et al 2015; Mollalign et al, 2015). It can also refer to Pre-XDR or XDR or pre-XDR and XDR combined.

- The authors should define rifampicin resistant and multi-drug resistant TB in the methods section

Authors’ response: Thank you. This is included in methods section of the revised manuscript. The rifampicin resistance (RR) includes any resistance to rifampicin, in the form of mono-resistance, poly-resistance, MDR or XDR. The MDR is a resistance to at least both isoniazid and rifampicin.

- The failure to include ART as a variable in the risk factor analysis for patients with HIV is problematic. If possibly I would strongly suggest that the analysis be reviewed with this risk factor as it has major implications for mortality of patients with RR/MDR-TB and HIV. ART status should be defined as on ART, not on ART or ART status not known.

Authors’ response: Thank you for your concern. Unfortunately, from among the studies reporting the link between HIV-infection and mortality, only one study also correlate ART status with death. For this reason, we were not able to do this as the data was not reported by studies we included.

- Definition of treatment outcomes should be included (i.e. how were success, lost to follow-up and failure defined in the included studies).

Authors’ response: Even though our outcome of interest was death and its risk factors, we included the treatment outcome definitions as per your suggestion in methods section of the revised manuscript.

Results
- Table 2: Insufficient detail provided on the standard treatment regimens used in the various studies. There has been rapid evolution since 2010 in the treatment of RR/MDR-TB and modifications in the treatment regimen could result in some of the heterogeneity in the results. Consider adding this data to table 2 (for example a column with % of patients treated with AG, % of patients treated with later-generation FQ and % of patients treated with BDQ for each study, if this data is available). If this level of detail is not available or not possible to retrieve, a narrative description of the regimens used in the included studies should be added to the methods section. They could be classified according to the WHO effectiveness categories in use at the time of the study (prior to the recent major change in WHO classification of second-line TB drugs).

Authors’ response: Thank you for these concerns. Almost all studies we included didn’t report proportion of patients who used AGs, later generation FQs and Bedaquiline. Except few, majority of the studies report specific drugs included in the SLD regimen. Accordingly, majority of the patients used cycloserine OR terizidone and/or clofazimine containing SLD regimen. SLD regimens containing one or both of these medicines are categorized by WHO as group B. Only two studies report SLD regimen used has added three drugs from levofloxacin or moxifloxacin, bedaquiline and linezolid under group A. DR-TB patients who participated in the remaining studies used standardized SLD regimen which were either not specified or not categorized as group A or B. A regimen of SLD containing medicines added to complete the regimen from injectable aminoglycosides (kanamycin, capreomycin, amikacin), ethambutol, pyrazinamide, ethionamide, para-amiosalicylic acid, delamanid, etc. are under group C. These narration was added to methods section of the revised manuscript and subgroup analysis was also performed to account for heterogeneity in mortality effects by these group of SLD regimen. However, there was no significant difference by the WHO grouping of SLD regimen used by the DR-TB patients. The mortality was 13% (95% CI: 10%-17%) for Group A, 15% (95% CI: 13%-17%) for group B, 19% (95% CI: 14%-24%) for group C and 21% (95% CI: 13%-29%) for standardized SLD regimen group not specified by the studies (S2 Fig). These values can give hints of reduced mortality rates for patients treated with group A and group B. This group of SLD regimen is part of evolution in DR-TB therapy in the last decade including addition of bedaquiline or delamanid, repurposed agents (clofazimine, linezolid) or later generation FQs (moxifloxacin, levofloxacin). These additions are made to Table 1 of the results section of the revised manuscript and narrated as per your recommendation.

- Table 2: should include % HIV prevalence and % on ART in each study. The study period for each included study should also be added,
- The proportion of patients with HIV on ART is not addressed in the results section and this has major implications for mortality.

Authors’ response: This is not a common variable to all studies included. Again, the studies which report HIV-coinfection as a factor linked with mortality didn’t also correlate ART status with death. Only one study (Schnippel et al 2015) categorize data by ART status. Thus, we were unable to address this because we have very limited control over the data as we are accessing only data which were published and presented in the studies.

Discussion

The discussion section needs to be re-written. The purpose of the discussion section is to highlight the findings of the study and place them in the context of the literature in the field. This was not done successfully. Instead the current discussion is a re-iteration of the results of the study without much additional insight or contextualisation. Many prominent recent articles in the field and important concepts are not highlighted.

Authors’ response: Thank you. We have highlighted our findings in the first paragraph of the revised manuscript. We also addressed contextualization of the findings in reference to set targets and previous meta-analyses reports. We have considered all relevant articles you recommended as well. Please check re-writing and modifications we made to our discussion.

The failure to discuss the impact ART on RR/MDR-TB treatment outcomes in people living with HIV stood out. Much has been written about this in the literature that is not cited or discussed.

Authors’ response: This requires a separate study because we didn’t plan at the beginning in the search for studies we included and they were not selected by the HIV and ART statuses. The studies included doesn’t categorize mortality by ART status. So, addressing it at this stage without pre-defined criteria doesn’t give a complete image of the impact of ART on DR-TB treatment.

A brief literature review revealed several relevant and important articles in this field that are not discussed or referenced suggesting that the authors have not performed an adequate literature review which may be why they appear to have difficulty contextualising their findings in the discussion.
- Bastos et al ERS 2017 - An updated systematic review and meta-analysis for treatment of multidrug-resistant tuberculosis
- Bisson et al Lancet 2020 - Mortality in adults with multidrug-resistant tuberculosis and HIV by antiretroviral therapy and tuberculosis drug use: an individual patient data meta-analysis
- Mesfin et al Plos One 2014 - Association between HIV/AIDS and Multi-Drug Resistance Tuberculosis: A Systematic Review and Meta-Analysis
- Khan et al. ERS 2017 Effectiveness and safety of standardised shorter regimens for multidrug-resistant tuberculosis: individual patient data and aggregate data meta-analyses
- Ahmad et al. The Lancet 2018. Treatment correlates of successful outcomes in pulmonary multidrug-resistant tuberculosis: an individual patient data meta-analysis
- Isaakidis et al IJTLD 2015. Treatment outcomes for HIV and MDR-TB co-infected adults and children: systematic review and meta-analysis.
- Fox et al. ERS 2017 Group 5 drugs for multidrug-resistant tuberculosis: individual patient data meta-analysis

Authors’ response: Thank you. All the articles are considered in the revised manuscript. Please check them in discussion section of the revised manuscript.

Yours sincerely,

Authors
