# Supplementary material for: Incidence and predictors of mortality among persons receiving second-line tuberculosis treatment in sub-Saharan Africa: A meta-analysis of 43 cohort studies
Source: PLoS One. 2021 Dec 10;16(12):e0261149. doi: 10.1371/journal.pone.0261149 (PMC8664218; doi:10.1371/journal.pone.0261149)
Supplement: Attachment — Submitted filename: PlosOne_Risk Factors of mortality_Review_v2.docx [file pone.0261149.s009.docx]

| **Section of the paper** | **Comment** |
| --- | --- |
| *General* | |
| Editing | Generally, the paper still requires extensive review and editing to ensure that proper grammar is used throughout. |
| *Title* | |
| Language and representativeness | The title was both concise and representative. Well done. |
| *Abstract* | |
| Introduction | According to the WHO 2020 report the rate of LTFU was higher than mortality as a final treatment outcome. However, if you are looking at all-cause mortality then your statement would be correct. You might just want to consider modifying the text slightly here to account for this. |
| Results | For this statement, you might want to include the RR’s  Individual studies also reported the increased risk of mortality in drug-resistant tuberculosis patients initiated with SLD after a month delay and those patients with tuberculosis treatment history. |
| *Background* | |
| General | Here I think you might mean rate and not report?  A high mortality report has also been found in DR-TB patients with undernutrition and excessive alcohol use [10].  This sentence does not really make sense:  A high mortality rate persisted for several years among DR-TB patients treated by a standardized SLD regimen regardless of our knowledge of the above factors [12].  Remove the period before the citations here:  However, the treatment success rate remains unacceptably low. The average annual mortality rate in resource-limited settings mirror the average global mortality despite a few resource-limited countries reaching a mortality rate of 30 percent or above. [2, 7, 23]. |
| *Methods* | |
| Eligibility criteria | I recommend that you simplify these sentences. They can be combined and edited for improved flow.  “Again, researchers excluded studies without separate data for MDR-TB treatment outcomes in the case of studies that also included patients with extensively drug-resistant (XDR) TB. Moreover, studies with treatment outcomes for pre-extensively drug-resistant (pre-XDR) or XDR-TB were also excluded. Researchers excluded studies with XDR-TB patients’ data as it can result from resistance to any fluoroquinolone or at least one of the three injectable aminoglycosides (capreomycin, kanamycin, and amikacin)[27] not to complicate the outcome observed for standardized SLD therapy.  I wonder if the authors only included studies which used the WHO definitions of outcomes.” |
| Covariate definition | How was alcohol misuse categorized? |
| Outcomes definition | If these outcome definitions are in line with the WHO outcome definitions, you can just mention this and provide a citation for the WHO definitions. Then you can further classify what you considered successful vs not successful and provide your definition for all cause mortality. |
| *Results* | |
| Risk Factors of Mortality | You report on HIV and comorbidities separately. You might just want to specify what these other comorbidities are…….. HIV is considered a co-morbidity. So in some ways these categorizations could be problematic. |
| *Discussion* | |
| General | Remove by analysis:  The pooled estimate of mortality proportion among the DR-TB patients treated with SLD in SSA was 17% by analysis.  Further editing is required throughout these sections for improved grammar and clarity. |
| *Limitations* | |
| General comments | Consider adding a point about the potential of there being an overlap of included patients in the SA studies. |
