# Supplementary material for: Incidence and predictors of mortality among persons receiving second-line tuberculosis treatment in sub-Saharan Africa: A meta-analysis of 43 cohort studies
Source: PLoS One. 2021 Dec 10;16(12):e0261149. doi: 10.1371/journal.pone.0261149 (PMC8664218; doi:10.1371/journal.pone.0261149)
Supplement: Attachment — Submitted filename: Response to Reviewers.docx [file pone.0261149.s010.docx]

**Authors’ Response to Reviewers’ comments**

Dear editor,

Thank you for this opportunity to revise our manuscript. We have addressed all the comments raised by the reviewers and incorporated them in the current revision. We also indicated our responses to each of the comments. Please look into the revised manuscript and response to reviewers’ comments for details.

PONE-D-20-25643R1

Incidence and predictors of mortality among persons receiving second-line tuberculosis treatment in sub-Saharan Africa: A meta-analysis of 43 cohort studies

PLOS ONE


Journal Requirements:

Please review your reference list to ensure that it is complete and correct. If you have cited papers that have been retracted, please include the rationale for doing so in the manuscript text, or remove these references and replace them with relevant current references. Any changes to the reference list should be mentioned in the rebuttal letter that accompanies your revised manuscript. If you need to cite a retracted article, indicate the article’s retracted status in the References list and also include a citation and full reference for the retraction notice.

**Authors’ response**: Thank you for this concern. Our reference list is complete and correct. And, to our knowledge, we don’t have any retracted paper to disclose.

Reviewers' comments:

**Reviewer #1**: Kindly review all of my comments in the attached file. Generally, I feel as though this manuscript still requires heavy editing for improved English language and clarity.

| **Section of the paper** | **Comment** |
| --- | --- |
| *General* | |
| Editing | Generally, the paper still requires extensive review and editing to ensure that proper grammar is used throughout.  **Authors’ response**: Thank you for this concern. We have made heavy editing to the current revised manuscript across. Please check the changes made throughout the manuscript to improve clarity alongside correcting grammatical errors. |
| *Title* | |
| Language and representativeness | The title was both concise and representative. Well done. |
| *Abstract* | |
| Introduction | According to the WHO 2020 report the rate of LTFU was higher than mortality as a final treatment outcome. However, if you are looking at all-cause mortality then your statement would be correct. You might just want to consider modifying the text slightly here to account for this.  **Authors’ response:** Thank you, this comment is well accepted and modified accordingly. As the studies identified didn’t specifically report the cause of death, the all-cause mortality is a realistic terminology that must be represented. Please check this modification in the background section of the abstract and introduction section of the current revision. |
| Results | For this statement, you might want to include the RR’s  Individual studies also reported the increased risk of mortality in drug-resistant tuberculosis patients initiated with SLD after a month delay and those patients with tuberculosis treatment history.  **Authors’ response**: Thank you so much. This is addressed and included in results section of the abstract in the current revision. |
| *Background* | |
| General | Here I think you might mean rate and not report?  A high mortality report has also been found in DR-TB patients with undernutrition and excessive alcohol use [10].  **Authors’ response**: Yes, this lack of clarity is corrected in the current revision.  This sentence does not really make sense:  A high mortality rate persisted for several years among DR-TB patients treated by a standardized SLD regimen regardless of our knowledge of the above factors [12].  **Authors’ response**: Thank you for your thorough evaluation and this sentence is removed from the current revision in which we aimed to avoid confusion in the document.  Remove the period before the citations here:  However, the treatment success rate remains unacceptably low. The average annual mortality rate in resource-limited settings mirror the average global mortality despite a few resource-limited countries reaching a mortality rate of 30 percent or above. [2, 7, 23].  **Authors’ response**: Thanks, this is edited as per your suggestion. |
| *Methods* | |
| Eligibility criteria | I recommend that you simplify these sentences. They can be combined and edited for improved flow.  “Again, researchers excluded studies without separate data for MDR-TB treatment outcomes in the case of studies that also included patients with extensively drug-resistant (XDR) TB. Moreover, studies with treatment outcomes for pre-extensively drug-resistant (pre-XDR) or XDR-TB were also excluded. Researchers excluded studies with XDR-TB patients’ data as it can result from resistance to any fluoroquinolone or at least one of the three injectable aminoglycosides (capreomycin, kanamycin, and amikacin) [27] not to complicate the outcome observed for standardized SLD therapy.  I wonder if the authors only included studies which used the WHO definitions of outcomes.”  **Authors’ response**: Thank you for this concern. As you well know, classifications of TB based on drug-resistance are not all mutually exclusive. We actually considered studies that define outcomes as per the WHO outcome definitions. But, persons receiving the SLD therapy for XDR-TB might have a more complicated clinical characteristics than that of the persons receiving SLD treatment for RR-TB/MDR-TB. Accordingly, we thought treating them equally as a similar population can potentially complicate the measurement of the drug-resistance TB treatment outcome. Again, we assumed that these people were one or more steps ahead as they already faced any form of resistance to fluoroquinolones or to at least one of the three injectable aminoglycosides. These were the reasons why we excluded the persons receiving the SLD therapy for XDR-TB treatments. |
| Covariate definition | How was alcohol misuse categorized?  **Authors’ response**: In our review, one of the study categorized alcohol use as ‘addicted’ and ‘not addicted’ while the other publication classified it as ‘alcohol use’ and ‘non-use’ as the predicting factor of mortality. This means that the studies didn’t have clear categorization and definition for the alcohol uses that includes misuses. They simply reported its association with the mortality outcome. |
| Outcomes definition | If these outcome definitions are in line with the WHO outcome definitions, you can just mention this and provide a citation for the WHO definitions. Then you can further classify what you considered successful vs not successful and provide your definition for all-cause mortality.  **Authors’ response**: Thank you very much for your guidance. These comments are well accepted and addressed in outcomes definition subsection of the methods section in the current revision. |
| *Results* | |
| Risk Factors of Mortality | You report on HIV and comorbidities separately. You might just want to specify what these other comorbidities are…….. HIV is considered a co-morbidity. So in some ways these categorizations could be problematic.  **Authors’ response**: Thank you for the concern. We addressed this by combining studies that report the presence of HIV-coinfection and other comorbidities together as ‘presence of HIV and other comorbidities’. The comorbidities reported included diabetes mellitus, myocardial infractions, hypertension, congestive heart failure, asthma, epilepsy, depression, chronic pulmonary insufficiency and pulmonary fibrosis. Again, we also added newer clinical conditions diagnosed in the patients as ‘diagnoses of other clinical conditions’ and this involved anemia, underweight (BMI < 18.5 Kg/m2), pneumothorax, pneumonia, hemoptysis, opportunistic infections, cavitary changes and nutritional problems. Accordingly, the incidences of mortality for these conditions were added and resulted in some changes to the RR estimates made. All these modifications are done in response to your comments and that of the other reviewer. Please check the results section of the current revision. |
| *Discussion* | |
| General | Remove by analysis:  The pooled estimate of mortality proportion among the DR-TB patients treated with SLD in SSA was 17% by analysis.  Further editing is required throughout these sections for improved grammar and clarity.  **Authors’ response**: Thank you very much. The current version is well edited throughout for the improved English language with the help of a native English speaker we have acknowledged. Please check the revised manuscript through for details of the changes made to improve the required grammar and clarity. |
| *Limitations* | |
| General comments | Consider adding a point about the potential of there being an overlap of included patients in the SA studies.  **Authors’ response**: Thank you so much. This comment is well accepted and added to the limitation section of the current revision. |

Reviewer #3: The present is an interesting paper.

Some issues.
Abstract should be shortened: e.g. introduction should be more focused on resistance to tuberculosis.
Abstract>primary end point should be defined
Abstract; it should be better specified how (And if) RRs were pooled
Abstract: some incidence or percentages should be added

**Authors’ response**: Thank you very much for your valuable comments. These comments are well accepted and addressed in each sub-sections as per your recommendation. The RRs were pooled for all statistically significant predictors that the studies reported. However, each of the individual predictors reported were incorporated to our model of pooling together if at least three studies had consistently reported the predictor of interest. Again, incidences of mortality in the presence of HIV-infection and other comorbidities were added together for a pooled RR. By the same token, incidences of newer clinical conditions diagnosed were added for our model of analysis. Some of these modifications are partly made in response recommendation of the other reviewer. Please check the modifications made in results section of the current revision.

Methods: due to observational design of the studies probably random effect should be used
Methods/results: comparison between different length of therapy is not inferential, but just observational. this should be clearly stated

**Authors’ response**: Thank you for these concerns. Yes, this was not inferential and it was the observed patient features. However, we compared incidence proportion of mortality by the median time period of follow-up for SLD therapy in an attempt to explore the sources of heterogeneity we found. This included other patient features such as the SLD treatment group they used and the setting (region) from where the patients participated in the study. Indeed, we found no homogenous group by these subgroupings, but we considered them in our explanation to indicate just what we did to the readers. These issues are clearly stated in methods and results section of the current revision.

Methods: authors should evaluate if they need to pool together RR (see PMID: 22360945)

**Authors’ response**: Thank you so much. We learned a lot from this reference and we made some modifications to our analysis in the estimates for RRs of predictors by adding incidences of mortality for some of the characteristics as indicated in the above response. Finally, we included the suggested article in our reference list for we referred it on how to add the incidences of mortality by specific predictors. Please check this in methods and results section of the current revised manuscript.

Yours sincerely,

Authors
